# HLA-DQ7 De Novo Donor-Specific Antibodies Are Associated with Increased Risk of Chronic Lung Allograft Dysfunction After Lung Transplantation

**DOI:** 10.3390/jcm15041608

**Published:** 2026-02-19

**Authors:** Maximilian Vorstandlechner, Julia Walter, Christian P. Schneider, Nicole Samm, Sebastian Michel, Paola Arnold, Roland Tomasi, Andrea Dick, Teresa Kauke

**Affiliations:** 1 Division of Thoracic Surgery, LMU University Hospital, Ludwig-Maximilians-Universität (LMU) München, 81377 Munich, Germany; julia.walter@med.uni-muenchen.de (J.W.); christian.schneider@med.uni-muenchen.de (C.P.S.); nicole.samm@med.uni-muenchen.de (N.S.); teresa.kauke@med.uni-muenchen.de (T.K.); 2 Department of Medicine V, LMU University Hospital, Ludwig-Maximilians Universität (LMU) München, 81377 Munich, Germany; 3 Transplantation Center Munich (LMU), LMU University Hospital, Ludwig-Maximilians-Universität (LMU) München, 81377 Munich, Germany; 4 Comprehensive Pneumology Center Munich (CPC-M), German Center for Lung Research (DZL) = Deutsches Zentrum für Lungenforschung, 81377 Munich, Germany; 5 Department of Cardiac Surgery, LMU University Hospital, Ludwig-Maximilians-Universität (LMU) München, 81377 Munich, Germany; 6 Department of Anesthesiology, LMU University Hospital, Ludwig-Maximilians-Universität (LMU) München, 81377 Munich, Germany; roland.tomasi@med.uni-muenchen.de; 7 Laboratory for Immunogenetics, Division of Transfusion Medicine, Cell Therapeutics and Haemostaseology, LMU University Hospital, Ludwig-Maximilians-Universität (LMU) München, 81377 Munich, Germany; andrea.dick@med.uni-muenchen.de

**Keywords:** lung transplantation, CLAD, dnDSA, HLA

## Abstract

**Background/Objectives**: Chronic lung allograft dysfunction (CLAD) remains the leading cause of late graft failure after lung transplantation (LuTX). De novo donor-specific anti-HLA antibodies (dnDSA), especially HLA-DQ, have been implicated; we assessed associations between dnDSA (class and specificity) and CLAD after LuTX. **Methods**: We retrospectively analyzed all LuTX recipients transplanted from 2005–2018 at a single center (n = 585). dnDSA were measured by Luminex single-antigen bead assays (MFI > 1000) at 1, 3, 6, and 12 months and at least annually thereafter. CLAD was defined by ISHLT criteria; time-to-event comparisons used log-rank testing. **Results**: dnDSA developed in 151/585 recipients (25.8%), predominantly class II (129/585; 22.1%); class I dnDSA occurred in 52/585 (8.9%). CLAD occurred more frequently in dnDSA-positive than dnDSA-negative recipients (64/151; 42.4% vs. 109/434; 25.1%; *p* < 0.0001). Rejection-attributed death was higher in dnDSA-positive recipients (19/151; 11.3% vs. 25/434; 5.3%; *p* = 0.01). Both class I and class II dnDSA were associated with higher CLAD rates (log-rank *p* < 0.001 each). Locus-specific analyses identified HLA-DQ dnDSA as strongly associated with CLAD (*p* < 0.0001); DQ7 was the most frequent specificity (n = 44) and showed the strongest association (*p* < 0.0001). **Conclusions**: dnDSA after LuTX were associated with increased CLAD incidence and rejection-attributed mortality, with a prominent association for HLA-DQ—particularly DQ7.

## 1. Introduction

For patients with end-stage lung disease (ELD), lung transplantation (LuTx) is a life-saving therapy [1]. Advances in surgical techniques, perioperative management and standardized immunosuppressive protocols have progressively reduced the incidence of post-transplant complications over recent decades, while markedly improving early postoperative survival [2]. Nevertheless, beyond infectious complications attributable to the necessitated immunosuppressive regimen, long-term survival remains largely constrained by chronic lung allograft dysfunction (CLAD) [3,4]. CLAD is defined by a sustained decline in allograft function and encompasses both obstructive and restrictive phenotypes, consequently making it the leading cause of late graft failure [3,5,6]. Once established, CLAD is often progressive and only incompletely reversible, translating into substantial morbidity, reduced quality of life and increased healthcare utilization. Accordingly, strategies that improve early risk stratification and enable prevention or earlier intervention are of major clinical interest.

Antibody-mediated alloimmune injury in LuTX is clinically captured under the umbrella term of antibody-mediated rejection (AMR) [7]. In 2016, the International Society for Heart and Lung Transplantation (ISHLT) issued a consensus report proposing diagnostic criteria for pulmonary AMR, centered on the detection of antibodies directed against donor human leukocyte antigens (HLA), compatible histopathology (with or without complement/C4d staining) and the exclusion of alternative causes of allograft dysfunction [8]. While this framework has been pivotal, it also underscores persistent challenges: AMR in the lung is heterogeneous, diagnostic certainty varies and the therapeutic relevance of subclinical AMR (de novo donor-specific antibodies [dnDSA] in the absence of overt allograft dysfunction) remains debated. In clinical practice, dnDSA may be detected during protocolized surveillance or triggered testing and may precede overt functional decline, but the subsequent course is variable. Some dnDSA may resolve or remain low-level, whereas others persist and coincide with inflammatory or fibrotic trajectories, contributing to uncertainty regarding when antibody detection should prompt intensified monitoring or therapeutic escalation. Within the multifactorial pathogenesis of chronic graft rejection, early observational studies associated post-transplant donor-directed HLA antibodies with inferior long-term allograft outcomes, including bronchiolitis obliterans syndrome (BOS) [9]. Accordingly, accumulating evidence implicates humoral alloimmunity, particularly the development of dnDSA, as a clinically important determinant of post-transplant outcomes [10,11,12]. Notably, a recurring signal across cohorts is that the clinical relevance of dnDSA appears to depend not only on their presence but also on qualitative features such as HLA class, locus, and specificity, which may reflect underlying immunogenic mismatch patterns and differing pathogenic potential.

Despite these developments, several key gaps remain. First, dnDSA screening strategies, assay interpretation and reporting practices are heterogeneous, limiting cross-study comparability and complicating the translation of dnDSA features, especially regarding kinetics and signal strength, into standardized clinical decision-making [13]. This heterogeneity includes differences in surveillance intensity, positivity thresholds, and how borderline or fluctuating reactivity is interpreted across centers. As a consequence, clinically meaningful subgroup signals may be difficult to compare directly between studies, and the implications of dnDSA detection for patient management remain inconsistently defined. Second, although HLA class-II-directed dnDSA have previously been repeatedly suspected and finally identified as a high-risk marker for the onset of CLAD in contemporary cohorts, it remains unclear which specificities are most strongly associated with chronic dysfunction and whether distinct targets delineate recipients at particularly high risk [14,15]. If specificity-level differences exist, they could provide a pragmatic next layer of stratification beyond dnDSA positivity alone and may help identify recipients in whom closer surveillance or earlier diagnostic evaluation for alloimmune injury is warranted. Third, as epitope- and allele-level risk models gain momentum, independent validation in real-world LuTX-cohorts is needed to establish clinical utility and generalizability [16,17].

Therefore, we investigated the association between dnDSA and CLAD after LuTX, with a specific focus on formation of dnDSA and assessed whether distinct HLA specificities confer differential risk of CLAD, building on previous evidence suggesting that anti-HLA class II antibodies are particularly deleterious in this setting. By examining locus- and specificity-level patterns, we aimed to move beyond the class I/class II dichotomy toward a more granular description of humoral alloimmune risk that may inform future prospective validation and clinically actionable monitoring strategies.

## 2. Materials and Methods

In this retrospective cohort study, we included patients who underwent LuTx at the division of thoracic surgery, LMU University Hospital, Munich, Germany, between 2005 and 2018. The study was approved by the institutional ethics committee (approval no. UE No. 22–0166), conducted in accordance with the Declaration of Helsinki and applicable institutional ethical standards. Medical records were reviewed retrospectively in order to collect demographic data, including age at the time of transplantation and sex, as well as the underlying diagnosis prior to the transplantation. Post-transplant follow-up data were reviewed to capture clinically relevant events and outcomes during routine outpatient surveillance and inpatient encounters, including graft dysfunction episodes and the development of CLAD as defined below. During the study period, institutional post-transplant management followed a standardized maintenance immunosuppression approach consisting of triple therapy with prednisolone, mycophenolate mofetil, and tacrolimus. This regimen was not changed throughout the entire study period. Induction therapy was not routinely used in this cohort. Routine post-transplant follow-up at our center comprised scheduled clinical assessments with lung function testing and additional diagnostics as clinically indicated, providing the basis for longitudinal outcome ascertainment in this retrospective analysis.

### 2.1. CLAD

The primary endpoint of the study, CLAD, as described in the ISHLT-consensus report, was defined as a continuous decrease in forced expiratory volume in the first second (FEV_1_) of at least 20% from the patient’s post-transplant baseline, as determined by averaging the two highest FEV_1_ values obtained at least three weeks apart [18]. An irreversible reduction in FEV1 to less than 80% of the baseline and an irreversible decline in total lung capacity (TLC) to less than 90% of baseline were considered indicators of restrictive allograft syndrome (RAS). In our cohort, the phenotypes BOS and RAS were referred to as CLAD. Exclusion of other possible confounders of deteriorating lung function such as infection, acute rejection, structural anomalies or mechanical obstacles was mandatory [19].

### 2.2. HLA-Typing

Routine HLA genotyping was performed on peripheral blood specimens from both donors and recipients, targeting five loci (HLA-A, -B, -C, -DRB1, and -DQB1), reflecting the routinely available locus set used for donor–recipient assessment during the study period. All typings were carried out at the Laboratory for Immunogenetics, LMU University Hospital Munich, Munich, Germany, using a Luminex-based sequence-specific oligonucleotide platform (LABType™ SSO Typing Kits, One Lambda, Inc., Canoga Park, CA, USA). Typing and reporting followed established laboratory quality assurance procedures and manufacturer guidance and the resulting donor–recipient locus assignments were used to support antibody specificity attribution and subsequent locus-specific analyses. As a limiting factor, comprehensive high-resolution data was not uniformly available in the earlier study years and retrospective high-resolution genotyping was not feasible for all recipients and donors because of limited archived sample material.

### 2.3. HLA Antibodies

HLA antibody testing was performed using a Luminex-based screening platform in conjunction with single-antigen bead (SAB) assays (LABScreen™ and LABScreen™ Single Antigen Class I and Class II; One Lambda, Inc., Canoga Park, CA, USA). Screening assays and SAB testing were used to detect and characterize anti-HLA antibodies, and donor-specificity was assigned by relating SAB-defined specificities to donor HLA typing. Antibody strength was recorded as mean fluorescence intensity (MFI), and HLA antibodies were classified as present when MFI exceeded 1000, consistent with the study definition for dnDSA positivity. Surveillance for dnDSA development was protocolized at one, three, six and twelve months during the first post-transplant year and continued at least annually thereafter as part of routine follow-up. Additional, non-scheduled testing was performed at the discretion of the treating team when clinically indicated—most commonly during evaluation of suspected AMR, new or progressive allograft dysfunction, or suspected CLAD—to complement clinical, functional, and radiologic assessment. This combined protocol-driven and clinically triggered approach aimed to capture both early dnDSA emergence and later antibody development during long-term follow-up. To evaluate dnDSA dynamics, we classified dnDSA as persistent when antibody positivity was sustained over a period of six months. Additionally, we categorized dnDSA as early-onset when first detected within the first 90 days after transplantation.

### 2.4. Statistical Analysis

R version 1.3.1093 (Free Software Foundation, Boston, MA, USA) was used for all statistical analyses. Baseline clinical and demographic variables were summarized and illustrated using descriptive statistics, tables and figures. For normally distributed data, continuous variables are displayed as mean ± standard deviation (SD). Non-normally distributed data, if applicable, are displayed as median and interquartile range (IQR)). The chi-square test (χ^2^) was used to compare categorical variables. Student’s *t*-test for normally distributed variables and the Mann-Whitney U test for non-parametric variables were used to compare two groups. Time-to-event analysis was performed by utilizing Kaplan-Meier curves and Log-rank-test to assess onset and timing of CLAD and the corresponding models handled censoring appropriately. Statistical significance was defined as *p* < 0.05.

### 2.5. Artificial Intelligence-Assisted Language Editing

During the preparation of this manuscript, the authors used ChatGPT version 5.2 (OpenAI, San Francisco, CA, USA) and NotebookLM (Google LLC, Mountain View, CA, USA) exclusively for language editing and stylistic refinement of the manuscript text. These tools were not used for data collection, data handling, statistical analysis, or interpretation of results. All content was reviewed and revised by the authors, who take full responsibility for the final manuscript.

## 3. Results

A total of 585 patients who underwent LuTX at our department between January 2005 and December 2018 were included in the analysis. During follow-up, 151 patients (25.8%) developed dnDSA, whereas 434 patients (74.2%) did not show evidence of donor-specific antibody formation. Baseline characteristics of both groups are summarized in Table 1.

Patients who newly developed DSA were younger at the time of transplantation compared to patients without antibody development (48.5 ± 13.5 years vs. 51.4 ± 15.1 years; *p* = 0.02). The proportion of bilateral transplantation was higher in the antibody-positive cohort (123/151; 81.5%) than in the non-antibody-group (315/434; 72.6%). There was no difference between groups with respect to distribution of sex distribution (female: 52/151 [34.4%] vs. 153/434 [35.3%]; *p* = 0.87) and underlying diagnosis leading to transplantation (Table 1, *p* = 0.06).

Regarding antibody characteristics, dnDSA were predominantly directed against HLA class II loci. HLA class II antibodies were detected in 129 patients, corresponding to 22.1% of the entire cohort, whereas class I antibodies were detected in 52 patients (8.9%). Frequencies of antibodies directed against individual loci and specificities are provided in Table 2.

**Figure 1:**
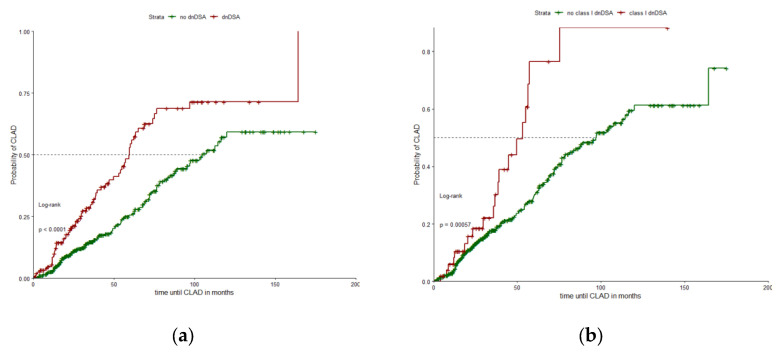

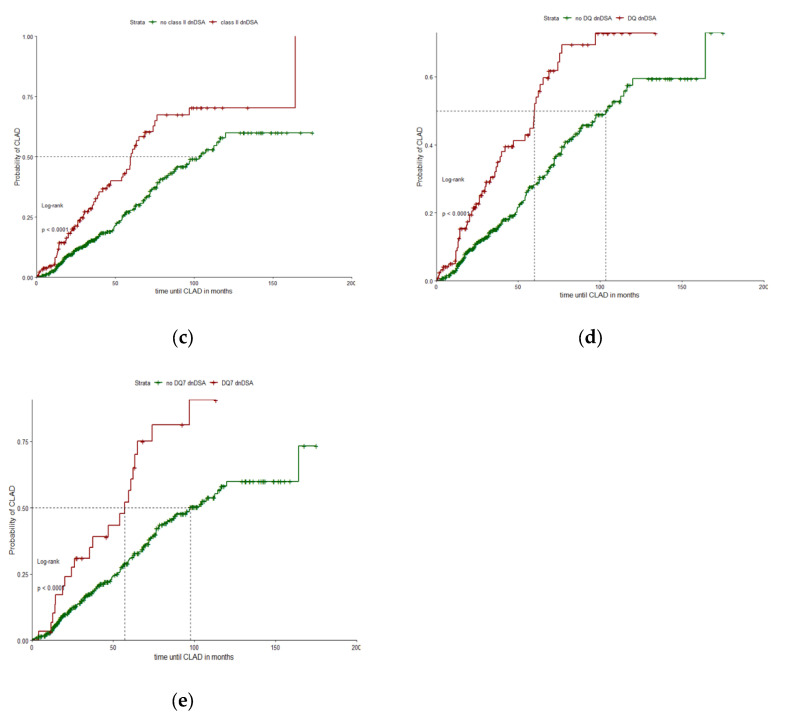
HLA class- and locus-specific probability of CLAD (**a**) dnDSA vs. non-dnDSA; (**b**) HLA-class I dnDSA vs. non-HLA-class I dnDSA; (**c**) HLA-class II dnDSA vs. non-HLA-class II dnDSA; (**d**) HLA-DQ dnDSA vs. non-HLA-DQ dnDSA; (**e**) HLA-DQ7 dnDSA vs. non-HLA-DQ7 dnDSA.

Time-to-event analyses demonstrated a significant association between dnDSA and the onset of CLAD. In the dnDSA-group, 64 of 151 patients (42.4%) developed chronic allograft dysfunction during follow-up, compared to 109 of 434 patients (25.1%) in the group without any signs of antibody-formation. This difference was significant in categorical comparison and in Kaplan-Meier analysis (χ^2^ test and log-rank test; *p* < 0.0001; Figure 1a). Transient dnDSA were observed in 37 recipients, among which 13 were classified as early-onset, occurring within the first 90 days after LuTX. In addition to the association with CLAD, analysis of causes of death showed differences between the groups. Death attributed to allograft rejection occurred significantly more frequently among patients with donor-directed antibodies (19/151; 12.6%) than among patients without antibody formation (25/434; 5.8%) (*p* = 0.01). Among recipients with CLAD, the frequency of restrictive phenotype was similar between those with dnDSA and those without dnDSA (7.7% vs. 8.5%, respectively).

To further evaluate whether the risk of CLAD differed according to antibody class, subgroup analyses were performed for HLA class I and class II antibodies. As illustrated in Figure 1b,c, class I and class II dnDSA were each associated with higher rates of CLAD-onset (log-rank: class I, *p* < 0.001; class II, *p* < 0.0001). These findings suggest that the increased risk of allograft dysfunction in patients subjected to dnDSAs was primarily not confined to one antibody class when analyzed at the class level. However, subsequently performed locus-specific analyses, aiming to identify whether particular antibody targets were to be considered independent risk factors for CLAD, locus-specific analyses did not identify any single HLA class I locus with a significant association (Table 2), despite the observed overall class-level association. In contrast, within HLA class II, a clear signal emerged for antibodies directed against the HLA-DQ locus, as patients with HLA-DQ dnDSA were at increased risk of developing chronically impaired graft function (Figure 1d; log-rank; *p* < 0.0001).

Given the prominence of HLA-DQ-directed antibodies, additional analyses were conducted to assess associations of specific -DQ targets with CLAD. Antibodies directed against HLA-DQ7 constituted the most frequent specificity observed in the cohort (n = 44) and were associated with CLAD (Figure 1e; log-rank; *p* < 0.0001). Beyond HLA-DQ7, antibodies directed against HLA-DQ6 showed a borderline association (*p* = 0.05). For antibodies directed against HLA-DQ2 and -DQ4, non-significant trends towards higher CLAD-rates were observed (*p* = 0.07 and *p* = 0.08, respectively). These subgroup findings indicate that, within the HLA-DQ locus, the association with a persistent decline in allograft function after transplant was not uniform across specificities and was most pronounced for DQ7 in this cohort.

## 4. Discussion

Although advances in surgical technique, perioperative care, and immunosuppression have improved early outcomes after LuTX, long-term survival remains predominantly limited by CLAD, the leading cause of late graft failure [3,5]. Within the multifactorial pathogenesis of CLAD, humoral alloimmunity, specifically donor directed antibodies, has increasingly been recognized as clinically relevant, both as a marker of alloimmune activation and as a potential contributor to chronic injury [10,11]. Against this background, we analyzed dnDSA after LuTX in a large single-center cohort (n = 585; January 2005 to December 2018) and examined whether antibody class and specificity are associated with differential CLAD risk.

DnDSA developed in 25.8% of recipients and were associated with a substantially higher incidence of CLAD during follow-up (42.4% vs. 25.1% in dnDSA-negative recipients; *p* < 0.0001). This is consistent with previous reports linking dnDSA to inferior CLAD-free survival and adverse graft outcomes after LuTX [11,15,20,21,22]. Beyond spirometric endpoints, dnDSA were also associated with a higher proportion of deaths attributed to allograft rejection, aligning with observations connecting dnDSA to graft-injury phenotypes, including AMR and chronic dysfunction [11]. Collectively, these findings support dnDSA as a clinically relevant post-transplant marker that co-segregates with subsequent long-term graft deterioration, although causal inference cannot be made from the present design.

Reported dnDSA incidence varies considerably across the literature, which is important when interpreting absolute rates. Protocolized surveillance cohorts have described higher cumulative incidences early after transplantation and during the first one to two years, with dnDSA frequently emerging in the early post-transplant period [10,23]. Differences across studies likely reflect heterogeneity in screening schedules, assay platforms, positivity thresholds (including MFI cutoffs) and definitions of persistence [20,24]. These methodological factors directly influence detection sensitivity and the proportion of transient signals captured as dnDSA. Nevertheless, across heterogeneous datasets, a consistent signal emerges: dnDSA are common after LuTX and repeatedly associate with CLAD and/or graft survival [20,22,25,26].

A central observation in our cohort is that dnDSA were dominated by HLA class II responses and specificity-level analyses pointed most clearly to HLA-DQ. This mirrors prior work suggesting that HLA-DQ-directed antibodies are disproportionately represented and clinically relevant after LuTX [14,15]. While both class I and class II dnDSA were associated with increased CLAD rates at the class level, locus-specific analyses did not identify a single class I locus driving the association, whereas a clear signal emerged for HLA-DQ within class II. This pattern suggests that class-level analyses may mask clinically meaningful heterogeneity at the locus level and that risk stratification may benefit from moving beyond the class I/class II dichotomy.

Within the HLA-DQ locus, our findings further suggest that the association with CLAD is not uniform across specificities. DQ7 was the most frequent DQ specificity and showed the strongest association with CLAD, whereas DQ6 showed a borderline association. One possible explanation for the prominence of DQ7 is that it may reflect underlying immunogenetic risk at the HLA-DQ locus rather than a purely allele-specific effect. Recent work by Kleid et al. highlights that high-risk HLA-DQ mismatch configurations and molecular (eplet) mismatch can predispose to the development of HLA-DQ dnDSA and are associated with adverse long-term outcomes after lung transplantation, including CLAD [27]. Accordingly, DQ7-directed dnDSA in our cohort may act as a marker of higher-immunogenic DQ mismatch patterns that promote sustained humoral alloimmunity. Importantly, the absence of statistical significance for other DQ specificities should not be interpreted as evidence of clinical irrelevance. Specificity-level analyses inherently involve smaller subgroups and fewer events, reducing statistical power and increasing the likelihood of type II error. In addition, multiple testing across specificities further warrants cautious interpretation. At the same time, genuine heterogeneity in the pathogenic potential of distinct DQ specificities cannot be excluded. The prominence of anti-HLA-DQ7 antibodies in our cohort is therefore noteworthy and supports the hypothesis that certain DQ targets may define a particularly high-risk phenotype. Clinically, this provides a pragmatic next layer of stratification, beyond dnDSA positivity alone, by incorporating whether antibodies are DQ-directed and which DQ specificity predominates.

Another clinically relevant dimension that could not be addressed is dnDSA persistence and antibody strength. Prior studies suggest that persistent dnDSA are associated with worse CLAD-free survival and higher AMR incidence compared with transient dnDSA [20]. In our cohort, dnDSA were defined using a positivity threshold (MFI > 1000). We were able to perform an additional exploratory assessment of dnDSA transience versus persistence; however, given the long study period beginning in 2005, longitudinal characterization was limited by heterogeneity and early-era follow-up testing was less consistently available. Accordingly, we did not evaluate peak MFI, MFI kinetics or complement-binding capacity. Incorporating dnDSA kinetics and persistence into future analyses will therefore be particularly important, especially for DQ-directed dnDSA.

From a management perspective, the lack of randomized controlled trials remains a major limitation and treatment strategies are still frequently extrapolated from other solid organ transplantation settings. Emerging observational evidence suggests that timing may be relevant: preemptive therapy for asymptomatic dnDSA has been associated with a lower risk of CLAD or death, whereas deferring treatment until clinically overt AMR has been linked to worse outcomes in some cohorts [2,28] This has supported risk-adapted approaches, ranging from IVIG-based strategies in selected patients to combination regimens for clinically manifest AMR [29,30]. Although we did not evaluate treatment effects, the strong association between DQ-directed (particularly DQ7) dnDSA and subsequent CLAD in our cohort suggests that these recipients represent a high-risk subgroup in whom closer follow-up and early evaluation of lung function decline may be warranted.

Several limitations should be considered when interpreting these findings. The retrospective single-center design introduces the possibility of unmeasured confounding and may limit generalizability to centers with different surveillance schedules, immunosuppressive strategies or thresholds for intervention. While the long study period allowed inclusion of a large cohort, it introduces temporal heterogeneity, including potential evolution and changes in clinical management and antibody testing practices over time. Although routine dnDSA surveillance followed an institutional schedule, additional clinically indicated testing may have varied, potentially influencing detection timing. High-resolution HLA genotyping was not uniformly available in the early years of the study, which limited allele- and epitope-level analyses. On another note, compared with prior CLAD phenotyping studies, the proportion of RAS in our cohort was lower and should be interpreted cautiously, given variability in phenotyping assessments over the long study period [31]. Given the retrospective observational design, the observed associations should be interpreted cautiously, as residual confounding cannot be fully excluded.

In summary, dnDSA formation after LuTX was associated with a substantially increased risk of CLAD and a higher proportion of rejection-attributed mortality. DnDSA were predominantly class II, and locus-specific analyses identified HLA-DQ as the central signal, with a particularly strong association for DQ7 in this cohort. These findings support the clinical value of dnDSA monitoring as part of post-transplant surveillance and suggest that specificity-level information—especially within HLA-DQ—may refine risk stratification beyond dnDSA status alone. Future work should integrate dnDSA kinetics, higher-resolution immunogenetics and injury biomarkers to improve early identification of high-risk trajectories and to inform prospective, evidence-based intervention strategies.

## Figures and Tables

**Table 1:** Patients’ characteristics (dnDSA vs. Non-dnDSA).

	dnDSA (n = 151)		Non-dnDSA (n = 434)		*p*
	Mean	SD	Mean	SD	
age at LuTX					
recipient	48.5	13.5	51.4	12.5	0.02
donor	45.3	15	45.3	15.8	0.98
	n	%	n	%	*p*
sex recipient					
male	86	57.0%	251	57.8%	
female	52	34.4%	151	34.8%	1.00
sex donor					
male	86	57.0%	254	58.5%	
female	65	43.0%	177	40.8%	0.74
condition					
CF	28	18.5%	69	15.9%	
COPD	32	21.2%	122	28.1%	
ILD	43	28.5%	130	30.0%	
other	48	31.8%	111	25.6%	0.24
type of surgery					
unilateral	28	18.5%	119	27.4%	
bilateral	123	81.5%	315	72.6%	0.04

Abbreviations: CF, cystic fibrosis; COPD: chronic obstructive pulmonary disease, dnDSA, de novo donor-specific antibodies; ILD; interstitial lung disease.

**Table 2:** DSA frequency and specificity.

	CLAD-Patients (n = 173)		Non-CLAD-Patients (n = 412)		
HLA	n	%	n	%	*p*
A1	3	1,9%	4	1.3%	0.44
A2	1	0.6%	3	1.0%	0.58
A3	2	1.3%	0	0.0%	0.11
A11	1	0.6%	1	0.3%	0.56
A23	1	0.6%	0	0.0%	0.34
A24	2	1.3%	2	0.7%	0.42
A25	1	0.6%	1	0.3%	0.56
A68	1	0.6%	0	0.0%	0.34
A69	1	0.6%	0	0.0%	0.34
B8	2	1.3%	3	1.0%	0.55
B27	0	0.0%	1	0.3%	0.66
B35	1	0.6%	0	0.0%	0.34
B44	3	1.9%	2	0.7%	0.22
B49	1	0.6%	0	0.0%	0.34
B56	1	0.6%	0	0.0%	0.34
B57	1	0.6%	1	0.3%	0.56
B61	1	0.6%	0	0.0%	0.34
C2	0	0.0%	1	0.3%	0.66
C3	1	0.6%	1	0.3%	0.56
C4	1	0.6%	0	0.0%	0.34
C5	0	0.0%	1	0.3%	0.66
C6	0	0.0%	1	0.3%	0.66
C7	1	0.6%	1	0.3%	0.56
C12	1	0.6%	0	0.0%	0.34
C15	0	0.0%	1	0.3%	0.66
DQ1	2	1.2%	0	0.0%	0.09
DQ2	17	9.8%	18	4.4%	0.08
DQ4	4	2.3%	2	0.5%	0.07
DQ5	4	2.3%	7	1.7%	0.42
DQ6	7	4.0%	6	1.5%	0.05
DQ7	24	13.9%	20	4.9%	<0.0001
DQ8	3	1.7%	8	1.9%	0.58
DQ9	2	1.2%	4	1.0%	0.57
DR4	1	0.6%	3	0.7%	0.66
DR7	3	1.7%	1	0.2%	0.08
DR8	0	0.0%	1	0.2%	0.70
DR13	0	0.0%	1	0.2%	0.70
DR14	1	0.6%	1	0.2%	0.50
DR15	0	0.0%	1	0.2%	0.70
DR51	2	1.2%	1	0.2%	0.21
DR53	2	1.2%	1	0.2%	0.21

## Data Availability

The data that support the findings of this study are available on request from the corresponding author. The data are not publicly available due to privacy or ethical restrictions.

## Abbreviations

The following abbreviations are used in this manuscript:

ACR	acute cellular rejection
AMR	antibody-mediated rejection
BOS	bronchiolitis obliterans syndrome
CF	cystic fibrosis
Chi^2^-test	χ^2^
CLAD	chronic lung allograft dysfunction
COPD	chronic obstructive pulmonary disease
dnDSA	de novo donor-specific antibodies
ELD	end-stage lung disease
FEV_1_	forced expiratory volume in the first second
HLA	human leukocyte antigen
ILD	interstitial lung disease
IQR	interquartile range
ISHLT	International Society for Heart and Lung Transplantation
IVIG	intravenous immunoglobulin
LuTX	lung transplantation
MFI	mean fluorescence intensity
RAS	restrictive allograft syndrome
SAB	single anigen bead
SD	standard deviation
TLC	total lung capacity
